# Diet Quality Trajectories and Musculoskeletal Health Among the Oldest Old: Findings from the Hertfordshire Cohort Study

**DOI:** 10.3390/nu18040569

**Published:** 2026-02-09

**Authors:** Elaine M. Dennison, Faidra Laskou, Harnish P. Patel, Nicholas Fuggle, Kate A. Ward, Gregorio Bevilacqua, Leo D. Westbury

**Affiliations:** 1 MRC Lifecourse Epidemiology Centre, University of Southampton, Southampton SO16 6YD, UK; 2 NIHR Southampton Biomedical Research Centre, University of Southampton and University Hospital Southampton NHS Foundation Trust, Southampton SO16 6YD, UK; 3 School of Biological Sciences, Victoria University of Wellington, Wellington 6012, New Zealand; 4 Academic Geriatric Medicine, University of Southampton, Southampton SO16 6YD, UK

**Keywords:** diet quality, dietary trajectories, musculoskeletal health, ageing, sociodemographic factors, community-dwelling older adults

## Abstract

**Background:** Few studies have examined changes in diet quality into old age, and related these changes to musculoskeletal outcomes. We examined this among Hertfordshire Cohort Study participants. **Methods:** In total, 178 individuals provided diet quality scores derived in 1998–2004, 2011 and 2017 (median age 64.0, 74.7 and 80.7) using principal component analysis of food frequency questionnaires; higher scores indicated healthier diets (more fruit and vegetables, oily fish and wholemeal bread, and less white bread, added sugar, full-fat dairy products, chips and processed meat). Pearson correlations between diet quality scores at each time-point were computed. Group-based trajectory modelling of diet quality scores was implemented; trajectory groups as predictors of musculoskeletal outcomes (history of hip/knee replacement, osteoporosis, fall in previous year, low grip strength, low gait speed) in 2017 were examined using logistic regression with age and sex included as covariates. **Results:** Diet quality showed moderate stability over time (0.64 < r < 0.74). Three trajectory groups were identified: low (29%), medium (51%), and high diet quality (20%). A higher diet quality group was related to greater odds (95% CI) of hip/knee replacement (1.85 (1.05, 3.26) per higher category); associations with other musculoskeletal outcomes were weak (*p* > 0.17). **Conclusions:** Weak associations were observed between diet quality trajectories and musculoskeletal outcomes. However, higher diet quality was related to increased likelihood of hip/knee joint replacement, potentially due to confounding by socioeconomic position. The stability of diet quality suggests individuals with poorer diets around age 65 are likely to maintain these patterns into old age and may benefit from targeted interventions.

## 1. Introduction

Nutritional status is well recognised as an important contributor to musculoskeletal health in later life [1,2,3]. In addition to the contribution of calcium intake, and vitamins including vitamin D and K to bone health [1,2], dietary protein intake is an important determinant of muscle mass in later life [3]. Malnutrition may be linked to inadequate calorific intake, which can result in being underweight and at higher risk of osteoporotic fracture [4], whilst dietary preference dominated by saturated fats and sugar may be linked to being overweight and obese, both of which are linked to poor bone health [4] and osteosarcopenic obesity [5]. Obesity is also a strong risk factor for the development and progression of osteoarthritis [6].

We have previously demonstrated associations between nutritional status and physical function in participants from a community-dwelling UK cohort, the Hertfordshire Cohort Study [7], and other research has linked diet quality with bone microarchitecture [8]. Qualitative work in the Hertfordshire Cohort Study has suggested that nutritional patterns change with ageing and may be driven in part by social and psychological factors [9]. Nutritional status is often inadequate even in community-dwelling older adults. In our own study, when sampled at mean age of 83 years, almost half (47%) of participants scored either ‘moderate’ (score 3–5) or ‘high’ (score ≥ 6) nutritional risk, using the DETERMINE nutritional risk checklist tool [7].

Previous studies have examined measures of diet quality in relation to conditions and health outcomes relating to musculoskeletal health. A recent systematic review of 22 observational studies (243,846 participants) on the association between high-quality dietary patterns and osteoporosis reported that high-quality dietary patterns were protective, with stronger associations observed among women [10]. In contrast, a 2024 systematic review of five studies examining dietary patterns in relation to risk of falls in adults reported inconsistent results, with findings which differed depending on the sex of participants and the study design (cross-sectional or longitudinal) [11]. A 2022 systematic review and meta-analysis of dietary patterns in relation to measures of skeletal muscle mass, muscle strength, muscle performance, and sarcopenia risk reported that higher adherence to a healthy dietary pattern was associated with a lower risk of gait speed reduction [12]. However, the association between adherence to a healthy dietary pattern and the other outcomes considered was not statistically significant. Previous research on the prospective associations between diet quality and joint arthroplasty is limited. However, a meta-analysis performed in 2022, comprising 15 studies (97,157 individuals), reported associations between a healthier diet and reduced risk of knee osteoarthritis [13].

Examining dietary patterns rather than individual nutrients offers several advantages. Dietary pattern analysis captures potential interactive effects of foods and nutrients consumed together, providing a more holistic understanding of the relationship between diet and disease outcomes [14]. This approach also reduces problems of collinearity among nutrients and more accurately reflects real-world eating behaviours and their health consequences. Among older adults, focusing on dietary patterns, rather than single nutrient intakes, may better represent changes in eating habits and overall diet composition that may occur with life transitions such as retirement, bereavement, or living alone. However, this approach also has limitations. The derivation of dietary patterns often depends on subjective analytical decisions, such as food item grouping and statistical methods, which can limit comparability between studies. Moreover, dietary patterns may obscure the influence of specific nutrients or foods, limiting understanding of biological mechanisms. Nonetheless, understanding dietary trajectories in later life can help identify critical periods for intervention, such as around retirement, support personalised nutrition strategies, and inform policies promoting healthy ageing.

Previous research has considered associations between dietary trajectories and musculoskeletal health, specifically physical performance [15]. However, this work was truncated at age 60–64 years. Overall, far fewer studies have examined dietary trajectories over long periods extending from around the age of retirement (age 65 years) into old age. Given the importance of nutrition to musculoskeletal health at older ages, we therefore evaluated the stability of diet quality in the Hertfordshire Cohort Study over a median of 17 years, from around the age of 65 up until the ninth decade. We also examined the sociodemographic correlates of dietary trajectories, and associations between these trajectories and adverse musculoskeletal outcomes. This information may be valuable to clinicians and policymakers as it may help us identify those individuals who are at highest risk of poor diet in later life, at a time when intervention might be easier and most beneficial.

## 2. Materials and Methods

### 2.1. The Hertfordshire Cohort Study

The Hertfordshire Cohort Study consists of 1579 men and 1418 women who were born in Hertfordshire (UK) from 1931–1939, and who still lived there in 1998–2004 when they completed a home interview and clinic visit for a detailed health assessment. At baseline (1998–2004), 966 participants from East Hertfordshire underwent a detailed musculoskeletal assessment. In 2011, 443/966 agreed to participate in a follow-up study, and 224 took part in an additional follow-up study in 2017. The analysis sample for this article comprised the 92 men and 86 women with measures of diet quality at all three time-points (1998–2004, 2011 and 2017). Participants may have dropped out of follow-up studies for several reasons, including death, serious illness that prevented participation, relocation, loss of contact, withdrawal of consent, or other personal circumstances. Additional information about the HCS has been published previously [16].

### 2.2. Assessment of Diet Quality in 1998–2004, 2011 and 2017

At baseline (1998–2004), diet was assessed using a food frequency questionnaire (FFQ), and principal component analysis was conducted using data from the full FFQ (129 food items) [17]. The 24 items with the largest absolute coefficients on the first principal component were selected to derive a diet quality score; these coefficients are presented in Supplementary Table S1. For each participant, the reported consumption of these 24 food items was standardized using the mean and standard deviation of the reported consumption at baseline, and then multiplied by the corresponding coefficient, and the resulting values were summed to obtain a diet quality score, with higher scores indicating healthier diets (higher consumption of fruit and vegetables, oily fish and wholemeal bread but lower consumption of white bread, added sugar, full-fat dairy products, chips and processed meat). The same coefficients for these 24 food items were applied to FFQs administered in 2011 and 2017, which included the same food items, to ensure that diet quality was assessed on a consistent scale across time-points. Previously, diet scores derived from the full and short FFQs at baseline were shown to be highly correlated (r = 0.912 in men; r = 0.904 in women) and to demonstrate similar associations with key nutrient intakes and with blood concentrations of vitamin C and lipids [17].

### 2.3. Ascertainment of Other Participant Characteristics in 1998–2004

Physical activity (Dallosso questionnaire [18]), alcohol consumption and smoking status were ascertained at the home interview by a clinician-administered questionnaire. Occupational social class was ascertained from the most recent or current full-time occupation for men and among women who never married, and from the husband’s occupation for ever-married women. Occupations were then classified according to the 1990 OPCS Standard Occupational Classification (SOC90) unit group for occupation [19]. Information on housing tenure (owned, mortgaged, rented or ‘other’) and the age at which participants left full-time education was also collected. Details were recorded of over-the-counter and prescription medications that participants were currently taking. These medications were coded into the following systems according to the British National Formulary: cardiovascular; respiratory; gastro-intestinal; endocrine; central nervous; malignant disease and immunosuppression; nutrition and blood; musculoskeletal and joint disease; eye; ear; nose; skin; miscellaneous; and genito-urinary tract. The number of systems medicated was then used as a marker of morbidity level.

At the baseline clinic, height (Harpenden pocket stadiometer, Chasmors Ltd., London, UK) and weight (SECA floor scale, Chasmors Ltd., London, UK) were measured and used to derive body mass index (BMI).

### 2.4. Ascertainment of Adverse Musculoskeletal Outcomes in 2017

In 2017, information was obtained through clinician-administered questionnaires and participants underwent scans at the MRC Elsie Widdowson Laboratory (Cambridge). Participants were asked to report current use of any over the counter and prescription medications. Participants were asked if a doctor had ever told them that they had osteoporosis. Measurement of aBMD was performed at both femoral necks using dual-energy X-ray absorptiometry (iDXA, GE-Lunar scanner, GE Healthcare, Madison, WI, USA); the lowest of the two readings was used in analyses. Participants with at least one of the following criteria were regarded as having osteoporosis: femoral neck bone mineral density T-score ≤ −2.5; current use of anti-osteoporosis medications (female hormone replacement therapy, bisphosphonates, raloxifene, or strontium); or self-reported diagnosis of osteoporosis by a doctor. Participants were also asked if they had fallen in the past year. Grip strength was assessed three times on each hand (Jamar dynamometer, Promedics, Blackburn, UK) using a standardised protocol; the highest of the six values was used for analysis. Low grip strength was defined according to the 2019 European Working Group on Sarcopenia in Older People (EWGSOP2) criteria as <27 kg for men and <16 kg for women [20]. Customary gait speed was assessed twice over an 8ft course; the mean time of two trials was used, and gait speed was calculated as distance (metres) divided by time (seconds). Low gait speed was defined as ≤0.8 m/s, consistent with EWGSOP2 criteria. Participants were also asked to report any operations that they had undergone; these free text responses were used to determine whether participants had ever had a hip/knee joint replacement.

### 2.5. Statistical Methods

Participant characteristics at the three time-points (1998–2004, 2011 and 2017) were described using summary statistics. Group-based trajectory modelling was implemented to identify distinct longitudinal trajectory groups of diet quality according to age, using the diet quality scores assessed at the three time-points [21]. Models with up to four latent trajectory groups were considered under the assumption of a censored normal distribution for the diet quality scores. To accommodate potential non-linear trends, all combinations of polynomial terms up to cubic order were considered for each group. Among the models where the highest order polynomial term was statistically significant (*p* < 0.05), the model with the lowest Bayesian Information Criterion was selected as the optimal model.

To examine baseline predictors of trajectory group membership, ordinal logistic regression models were fitted with the diet quality trajectory group as the outcome. Separate models were implemented for the following baseline characteristics: age; sex; BMI; ever smoked regularly (yes/no); high alcohol intake (>14 units/week versus ≤14 units); Dallosso physical activity score; age left full-time education (≥15 years versus <15 years); housing tenure (owned/mortgaged versus rented/other); occupational social class (non-manual versus manual); and number of systems medicated. To examine associations between the diet quality trajectory groups and later-life musculoskeletal outcomes, binary logistic regression models were used to estimate the odds of the following adverse outcomes ascertained at the 2017 follow-up: osteoporosis; falls in the previous year; low grip strength (<27 kg men, <16 kg women); low gait speed (≤0.8 m/s); and history of hip/knee replacement. Age and sex were included as covariates in all models. Stata (version 17.0) was used for statistical analysis. Men and women were pooled as sex-interaction effects were not statistically significant.

## 3. Results

### 3.1. Descriptive Statistics

Descriptive statistics for the participant characteristics at the three time-points (1998–2004, 2011 and 2017) are presented in Table 1. Median (lower quartile, upper quartile) ages at the three time-points were 64.0 (62.0, 66.4), 74.7 (73.0, 77.1) and 80.7 (78.9, 83.1) years. Mean (SD) diet quality scores were higher among women than men at the first (0.6 (1.1) versus −0.3 (1.2)), second (0.5 (1.4) versus −0.3 (1.5)) and third (0.7 (1.1) versus 0.2 (1.2)) time-point. Compared to participants who were not included in the current study, those who were included generally had better health behaviours regarding smoking and physical activity, and were more likely to have markers indicative of higher socioeconomic position, such as home ownership and a non-manual occupation (Supplementary Table S2).

### 3.2. Tracking of Diet Quality over Time

Pearson correlations between diet quality scores at the three time-points (1998–2004, 2011 and 2017) are presented in Table 2. Fairly strong pairwise correlations (0.64 < r < 0.74) were observed between diet quality scores at these three time-points, indicating stability of diet quality between 1998–2004 and 2017.

### 3.3. Trajectory Groups of Diet Quality

**Figure 1:**
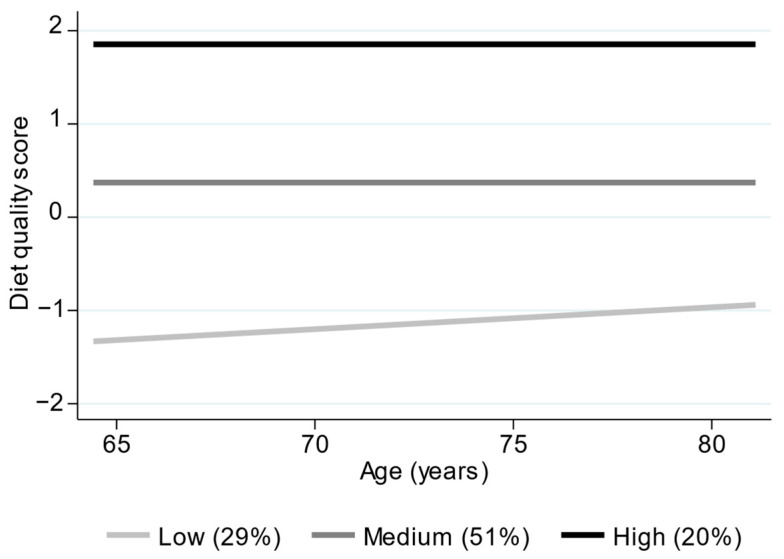
Diet quality trajectory groups according to age.

The optimum group-based trajectory model for diet quality according to age is presented in Figure 1. This model had three trajectory groups for diet quality (low (29%), medium (51%) and high (20%)). Differences in levels of diet quality between trajectory groups were considerably greater than differences in rates of change in diet quality. Baseline descriptive statistics, stratified by diet quality trajectory group, are presented in Supplementary Table S3.

### 3.4. Associations Between Baseline Characteristics and Diet Quality Trajectory Groups

Odds ratios (95% CI) for being in a higher diet quality trajectory group according to HCS baseline participant characteristics are presented in Table 3. The following characteristics were related to increased likelihood of being in a higher diet quality trajectory group: female sex (odds ratio (95% CI): 4.01 (2.14, 7.51)); high alcohol intake of >14 units/week (2.23 (1.00, 4.95)); leaving full-time education at age 15 years or later (2.52 (1.04, 6.15)); and non-manual occupational social class (1.97 (1.10, 3.54)). The association with female sex was adjusted for baseline age, and all other associations reported above were adjusted for baseline age and sex.

### 3.5. Associations Between Diet Quality Trajectory Groups and Adverse Musculoskeletal Outcomes at the 2017 Follow-Up

Odds ratios (95% CI) for adverse outcomes at follow-up per higher diet quality trajectory group are presented in Table 4 after adjustment for age at the 2017 follow-up and sex. Being in a higher diet quality trajectory group was related to increased risk of having a history of hip/knee replacement (1.85 (1.05, 3.26)). Associations for the remaining adverse outcomes were weak (*p* > 0.17).

## 4. Discussion

The findings from this study indicate that diet quality remained relatively stable over the nearly two-decade follow-up period. While this might be perceived as positive, it also suggests that individuals with poorer diets are unlikely to improve their diet and dietary patterns without intervention. Several baseline characteristics were associated with a greater likelihood of being in a higher diet quality trajectory group, including being female, higher consumption of alcohol, remaining in full-time education beyond age 15, and having a non-manual occupational social class. Notably, individuals in the higher diet quality trajectory groups also had an increased likelihood of reporting a history of hip or knee replacement at the 2017 follow-up, while associations with other adverse musculoskeletal outcomes were generally weak.

In our study, markers of higher socioeconomic position, such as remaining in full-time education for longer and a non-manual occupational social class, were associated with better diet quality. These findings align with extensive evidence demonstrating that social determinants of health, including education, income, and occupation, play an important role in shaping lifestyle behaviours and nutritional choices [22]. These disparities arise from social and structural determinants, including differences in food affordability and nutritional literacy, as well as broader influences such as neighbourhood food environments [22].

Our study found generally healthier diets in women, a pattern reported in other studies that used other nutritional indices [23]. Our cohort consisted of community-dwelling older adults, a factor that may have maintained apparent stability of nutritional choice as frailty is often linked to poorer nutritional status [24]. In our own study, we did not find associations between nutritional trajectory and musculoskeletal outcomes, other than joint arthroplasty. We hypothesize that this may be linked to a healthy cohort bias and limited power to study some associations; all our study participants were still living at home, and the prevalence of frailty was very low. However, we did find higher rates of joint replacement linked to higher diet quality. A limitation of our data is that we are unable to distinguish between private and publicly funded surgeries. In the UK, joint replacement has been linked to socioeconomic status, with those of greater material wealth being more able to afford to pay privately for such operations [25]. Therefore, it is plausible that our observed association between higher diet quality and increased likelihood of joint replacement is driven entirely by socioeconomic differences in access to surgery rather than any health impact of diet quality. Therefore, this finding should be interpreted with caution. Finally, we also observed higher alcohol intake in participants with higher nutritional patterns. This may also reflect confounding by higher socioeconomic class [26].

Within our cohort, we identified participants with poorer nutritional patterns. We were particularly interested in studying older adults living in their own homes, who still have autonomy over food choices. Nutritional choice is complex; it was the topic of a recent position piece, which suggested that there should be more responsibility placed on the government and food manufacturers rather than individuals [27], so any scalable interventions need to be multifactorial. For example, a nutritional risk screening programme, using a validated tool such as the Malnutrition Universal Screening Tool (MUST) or DETERMINE, could be implemented in the community as part of an annual assessment for older adults. This would enable early identification of individuals at high risk of malnutrition and allow provision of targeted support and health interventions to improve their nutritional status. At the individual level, we recently suggested that retirement is a time of transition when lifestyle modification might be possible, despite the challenge of long-term behaviours [28]. A healthy conversation skills intervention is one method that has been trialled in this cohort, with preliminary data suggesting it may be effective, even in later life [29]. Social factors have been identified as important contributors to dietary choice in this cohort and might be harnessed in any interventions.

Strengths of this study include the repeated assessment of diet across three time points spanning nearly two decades, the availability of a broad set of baseline characteristics and follow-up outcomes, and the rigorous phenotyping protocols applied within HCS. However, our study has several limitations. First, substantial selection and attrition biases may be present as the analytical sample comprised relatively healthy, community-dwelling older adults who remained engaged in the study over multiple follow-up stages. This ‘healthy participant’ effect likely reduces variability in both the dietary exposure and musculoskeletal outcomes, limiting statistical power and increasing the risk of type II errors. Moreover, participants were all Caucasian. Consequently, the generalisability of our findings may be much more limited for populations with greater socioeconomic deprivation, higher levels of frailty (such as nursing home residents), or those from different ethnic backgrounds or countries. Despite this, the baseline characteristics of the cohort were broadly comparable to those of participants in the nationally representative Health Survey for England. Second, baseline dietary assessment occurred between 1998 and 2004. Dietary environments, food availability, and public health recommendations have changed substantially since then, limiting the relevance of these findings for contemporary nutrition policy. Third, the sample size was small for trajectory modelling, particularly when outcomes are relatively infrequent. This constrains the precision of effect estimates and precludes robust subgroup or interaction analyses, such as sex-specific effects. This would have also reduced the statistical power to detect associations. However, the observed findings remain biologically plausible and consistent with previous research. Fourth, several musculoskeletal outcomes relied on self-report, including osteoporosis diagnosis and falls, introducing potential recall bias. In addition, for the osteoporosis outcome, participants who did not report doctor-diagnosed osteoporosis were assumed not to have the condition, even if their T-score or medication data were missing. However, the association between trajectory group membership and osteoporosis was similarly weak when repeated in the subsample of participants (155 of 179) with complete data for all three components used to define osteoporosis status. A further limitation is that self-reported doctor-diagnosed osteoarthritis was not collected at the 2017 follow-up; instead, we used history of hip or knee joint replacement as a proxy for severe osteoarthritis. This measure may not capture individuals with osteoarthritis who have not undergone arthroplasty, for example due to limited access to surgery.

## 5. Conclusions and Future Perspectives

In summary, we have reported generally stable diet quality trajectories over 17 years of follow-up in older adults, an observation that might be considered an opportunity or a challenge. We did not formally assess cognitive function in our study; our sample might be considered generally healthier than many of their peers, and cognitive impairment was not reported by participants or their carers. Future work should include participants who report memory problems, as this might be expected to be a group at high risk. Although we did not report strong associations with musculoskeletal outcomes in this sample, larger studies of older adults are now indicated, especially in populations where the prevalence of frailty is higher. Given demographic changes and increasing life expectancy, this work is urgently required.

In this study, we used observational data from a well-characterized cohort to examine diet quality trajectories in relation to sociodemographic and lifestyle factors, and in relation to musculoskeletal outcomes. While these analyses are descriptive, a key strength is the long duration of follow-up into very old age, a life stage for which high-quality dietary data are scarce, combined with detailed phenotyping. Although residual confounding is possible, the findings highlight population-level associations between socioeconomic and lifestyle factors around the time of retirement and dietary patterns nearly two decades later. These results suggest that interventions in the seventh decade of life may have the potential to yield long-lasting benefits extending into the oldest old.

## Figures and Tables

**Table 1:** Descriptive statistics of the analysis sample.

Participant Characteristic	Mean (SD); Median (Lower Quartile, Upper Quartile); or n (%)	
	Men (n = 92)	Women (n = 86)
**Baseline (1998** **–** **2004)**		
Age (years)	63.1 (61.7, 65.4)	65.1 (62.8, 67.2)
BMI (kg/m^2^)	26.5 (3.7)	26.4 (4.2)
Ever smoked regularly	55 (60%)	28 (33%)
High alcohol intake (>14 units per week)	35 (38%)	2 (2%)
Dallosso physical activity score	64 (54, 79)	64 (57, 71)
Diet quality score	−0.3 (1.2)	0.6 (1.1)
Age left full-time education (≥15 years)	77 (84%)	71 (83%)
Home ownership (owned/mortgaged)	80 (87%)	74 (86%)
Non-manual occupational social class	41 (47%)	42 (49%)
Number of systems medicated	1 (0, 1)	1 (0, 2)
**2011 follow-up**		
Age (years)	74.6 (73.1, 77.0)	74.9 (73.0, 77.2)
Diet quality score	−0.3 (1.5)	0.5 (1.4)
**2017 follow-up**		
Age (years)	80.6 (79.0, 83.0)	80.9 (78.7, 83.2)
Diet quality score	0.2 (1.2)	0.7 (1.1)
Osteoporosis	14 (15%)	34 (40%)
Fall in previous year	16 (17%)	12 (14%)
Low grip strength (<27 kg men, <16 kg women)	28 (31%)	22 (26%)
Low gait speed (≤0.8 m/s)	65 (71%)	65 (76%)
Hip or knee replacement	20 (22%)	16 (19%)

Osteoporosis: any of the following: femoral neck T-score ≤ −2.5; taking anti-osteoporosis medications; or self-reported osteoporosis diagnosis by doctor. Low grip strength and gait speed thresholds were based on the 2019 European Working Group on Sarcopenia in Older People (EWGSOP2) definition. The follow-up stage is stated in the column ‘Participant Characteristic’ in bold font.

**Table 2:** Pearson correlations between diet quality scores at the three time-points.

Time-Point	1998–2004	2011
2011	0.73	
2017	0.65	0.67

*p* < 0.001 for all associations.

**Table 3:** Odds ratios (95% CI) for being in a higher diet quality trajectory group according to HCS baseline participant characteristics.

HCS Baseline Characteristic	Adjusted for Baseline Age and Sex	
	Odds Ratio (95% CI)	*p*-Value
Age (per year increase)	0.97 (0.87, 1.08)	0.597
Sex (female)	4.01 (2.14, 7.51)	<0.001
BMI (per unit increase)	0.99 (0.92, 1.06)	0.727
Ever smoked regularly	0.94 (0.52, 1.68)	0.824
High alcohol intake (>14 units/week)	2.23 (1.00, 4.95)	0.049
Dallosso physical activity score (per unit increase)	1.00 (0.98, 1.02)	0.726
Age left education (≥15 years)	2.52 (1.04, 6.15)	0.042
Housing tenure (owned/mortgaged)	1.55 (0.69, 3.49)	0.288
Occupational social class (non-manual)	1.97 (1.10, 3.54)	0.023
Number of systems medicated (per unit increase)	1.14 (0.86, 1.51)	0.377

Unless indicated otherwise, odds ratios are presented for the presence versus absence of the HCS baseline characteristic. Associations for age were only adjusted for sex; associations for sex were only adjusted for age. Odds ratios were estimated using ordinal logistic regression models.

**Table 4:** Odds ratios (95% CI) for adverse musculoskeletal outcomes at the 2017 follow-up per higher diet quality trajectory group.

Musculoskeletal Outcome	Adjusted for Age at the 2017 Follow-Up and Sex	
	Odds Ratio (95% CI)	*p*-Value
Osteoporosis (from doctor-diagnosis, t-score or medications)	0.78 (0.45, 1.35)	0.374
Fall in previous year	1.17 (0.63, 2.14)	0.621
Low grip strength (<27 kg (men), <16 kg (women))	1.28 (0.77, 2.13)	0.341
Low gait speed (≤0.8 m/s)	0.70 (0.41, 1.18)	0.178
Hip or knee replacement	1.85 (1.05, 3.26)	0.033

Osteoporosis: any of the following: femoral neck T-score ≤ −2.5; taking anti-osteoporosis medications; or self-reported osteoporosis diagnosis by doctor. Odds ratios were estimated using binary logistic regression models.

## Data Availability

The data used in this article cannot be shared widely due to consent restrictions. The participants only consented for their data to be shared with the Hertfordshire Cohort Study Research Team. Requests to access Hertfordshire Cohort Study data for new research projects should be made to E.M.D. (emd@mrc.soton.ac.uk).

## Supplementary Materials

The following supporting information can be downloaded at: https://www.mdpi.com/article/10.3390/nu18040569/s1, Table S1: First principal component coefficient for each food item used to derive the diet quality score; Table S2: Comparison of baseline participant characteristics between the analysis sample and the group of participants who were not included in the analysis sample; Table S3: Participant characteristics at baseline (1998–2004), stratified by diet quality trajectory group.

## Abbreviations

The following abbreviations are used in this manuscript:

MRC	Medical Research Council
NIHR	National Institute for Health and Care Research
NHS	National Health Service
UK	United Kingdom
CI	Confidence Interval
FFQ	Food Frequency Questionnaire
BMI	Body Mass Index
aBMD	Areal Bone Mineral Density
EWGSOP2	Revised 2019 European Working Group on Sarcopenia in Older People
SD	Standard Deviation
MUST	Malnutrition Universal Screening Tool
