# Supplementary material for: Diet Quality Trajectories and Musculoskeletal Health Among the Oldest Old: Findings from the Hertfordshire Cohort Study
Source: Nutrients. 2026 Feb 9;18(4):569. doi: 10.3390/nu18040569 (PMC12942649; doi:10.3390/nu18040569)
Supplement: Supplementary file 1 [file nutrients-18-00569-s001.zip › nutrients-4069614-supplementary.pdf]

Supplementary Table 1: First principal component coefficient for each food item used to derive the diet quality score

	Food item	First principal component coefficient
Positive coefficients		
1.	Peppers (raw and cooked)	0.193
2.	Green salad (eg lettuce, cucumber)	0.182
3.	Garlic (raw and cooked)	0.175
4.	Tropical fruits (eg melon, pineapple, kiwi)	0.154
5.	White fish (cooked, not in batter)	0.153
6.	Marrow and courgettes	0.151
7.	Oily fish (eg mackerel, salmon)	0.149
8.	Pasta (eg spaghetti, macaroni)	0.145
9.	Yogurt (low-fat)	0.134
10.	Brown or wholemeal bread	0.124
11.	Apples	0.114
12.	Bananas	0.103
13.	Reduced fat milk	0.054
14.	Reduced fat spread	0.018
Negative coefficients		
15.	Eggs (boiled, scrambled)	-0.047
16.	Boiled and jacket potatoes	-0.086
17.	Bacon and gammon	-0.089
18.	Biscuits	-0.101
19.	Full fat milk	-0.119
20.	Meat pies	-0.126
21.	Full-fat spreading fat	-0.149
22.	Chips	-0.152
23.	Added sugar	-0.159
24.	White bread	-0.201

This table was adapted from the article *Robinson SM, Jameson KA, Bloom I, Ntani G, Crozier SR, Syddall H, Dennison EM, Cooper CR, Sayer AA. Development of a Short Questionnaire to Assess Diet Quality among Older Community-Dwelling Adults. J Nutr Health Aging. 2017;21(3):247-253. doi: 10.1007/s12603-016-0758-2. PMID: 28244562.*

Supplementary Table 2: Comparison of baseline participant characteristics between the analysis sample and the group of participants who were not included in the analysis sample

Participant characteristic at baseline (1998-2004)	Mean (SD); median (lower quartile, upper quartile); or n(%)	
	**Group included in analysis sample (n=178)**	**Group not included in analysis sample (n=2819)**
Age (years)	64.0 (62.0, 66.4)	66.3 (64.1, 68.3)
Sex (female)	86 (48%)	1332 (47%)
BMI (kg/m^2)	26.5 (3.9)	27.4 (4.4)
Ever smoked regularly	83 (47%)	1529 (54%)
High alcohol intake (>14 units per week)	37 (21%)	617 (22%)
Dallosso physical activity score	64 (57, 71)	57 (50, 71)
Diet quality score	0.1 (1.2)	0.0 (1.2)
Age left full-time education (≥15 years)	148 (83%)	2300 (82%)
Home ownership (owned/mortgaged)	154 (87%)	2231 (79%)
Non-manual occupational social class	83 (48%)	1130 (41%)
Number of systems medicated	1 (0, 2)	1 (0, 2)

Supplementary Table 3: Participant characteristics at baseline (1998-2004), stratified by diet quality trajectory group

Participant characteristic at baseline (1998-2004)	Mean (SD); median (lower quartile, upper quartile); or n(%)		
	Diet quality trajectory group		
	Low diet quality (n=51)	Medium diet quality (n=91)	High diet quality (n=36)
Age (years)	64.4 (61.7, 66.4)	63.9 (62.3, 66.1)	64.6 (62.6, 67.0)
Sex (female)	11 (22%)	51 (56%)	24 (67%)
BMI (kg/m^2)	25.9 (4.4)	27.2 (3.8)	25.5 (3.1)
Ever smoked regularly	27 (53%)	42 (46%)	14 (39%)
High alcohol intake (>14 units per week)	12 (24%)	17 (19%)	8 (22%)
Dallosso physical activity score	64 (50, 79)	64 (50, 71)	71 (57, 71)
Diet quality score	-1.3 (0.8)	0.3 (0.7)	1.6 (0.8)
Age left full-time education (≥15 years)	39 (76%)	76 (84%)	33 (92%)
Home ownership (owned/mortgaged)	43 (84%)	78 (86%)	33 (92%)
Non-manual occupational social class	19 (39%)	42 (47%)	22 (63%)
Number of systems medicated	1 (0, 1)	1 (0, 2)	1 (0, 2)
